# Experiences with carotid endarterectomy at Sree Chitra Tirunal Institute

**DOI:** 10.4103/0972-2327.42937

**Published:** 2008

**Authors:** Madathipat Unnikrishnan, Shivananda Siddappa, Rajesh Anto, Vivek Babu, Benny Paul, Thirur Raman Kapilamoorthy, Sivasubramanian Sivasankaran, Samavedam Sandhyamani, Rupa Sreedhar, Kuruppath Radhakrishnan

**Affiliations:** Department of Cardiovascular Surgery, Sree Chitra Tirunal Institute for Medical Sciences and Technology, Trivandrum, India; 1 Department of Imaging and Interventional Radiology, Sree Chitra Tirunal Institute for Medical Sciences and Technology, Trivandrum, India; 2 Department of Cardiology, Sree Chitra Tirunal Institute for Medical Sciences and Technology, Trivandrum, India; 3 Department of Pathology, Sree Chitra Tirunal Institute for Medical Sciences and Technology, Trivandrum, India; 4 Department of Anaesthesiology, Sree Chitra Tirunal Institute for Medical Sciences and Technology, Trivandrum, India; 5 Department of Neurology, Sree Chitra Tirunal Institute for Medical Sciences and Technology, Trivandrum, India

**Keywords:** Carotid artery, stenosis, stroke, carotid endarterectomy

## Abstract

**Background:**

Atherosclerotic carotid artery disease poses a grave threat to cerebral circulation, leading to a stroke with its devastating sequelae, if left untreated. Carotid endarterectomy has a proven track record with compelling evidence in stroke prevention.

**Objectives:**

a) To confirm that carotid endarterectomy (CEA) is safe and effective in preventing stroke at both short and long term. b) to demonstrate long term patency of internal carotid artery when arteriotomy repair is performed using autologous saphenous vein patch.

**Materials and Methods:**

During ten years, from September 1997 to February 2008, thirty nine patients who underwent consecutive carotid endarterectomy at our institute, form the basis of this report. Their age ranged from thirty to seventy eight years, with a mean age of 56. There were four women in this cohort. Thirty seven patients were symptomatic with >70% stenosis and two were asymptomatic with >80% stenosis, incidentally detected. Imaging included Duplex scan and MRA for carotid territory and brain, and non-invasive cardiac assessment. Co-morbidities included smoking, hypertension, diabetes, and coronary artery disease. Carotid Endarterectomy was performed under general anaesthesia, using carotid shunt and vein patch arteriotomy repair.

**Results:**

All the patients made satisfactory recovery, without major adverse cerebral events in this series. Morbidities included Transient Ischemic Attack (TIA) in two, needing only medications in one, and carotid stenting in the other. Minor morbidities included neck hematoma in two and transient hypoglossal paresis in three patients. Yearly follow-up included duplex scan assessment for all the patients. Two patients died of contralateral stroke, two of myocardial events and two were lost to follow up. Thirty three patients are well and free of the disease during the follow up of three to 120 months.

**Conclusion:**

Carotid endarterectomy provided near total freedom from adverse cerebral events and its catastrophic sequelae, leading to excellent outcome, both short as well as long term.

## Introduction

Carotid endarterectomy (CEA), first performed by Eastcott of England and perfected by Michael DeBakey, has saved millions around the world from the devastating sequelae of stroke. During the late 90s, the procedure underwent tight scrutiny and testing within a host of trials, unparalleled in the history of modern medicine, and proved its efficacy in stroke prevention. Carotid bifurcation stenosis, the major cause of ischemic stroke, is less prevalent in India and the Orient, in contrast to western world. With overwhelming and level I evidence, CEA as the standard of care to prevent stroke is gaining acceptance in our country, albeit at a slow pace

## Materials and Methods

During the period of 10 years from September 1997 to February 2008, 39 patients who underwent CEA in the vascular division of our tertiary care institute are included in this retrospective study. The patients who underwent CEA were identified from operation registers and data was collected from the medical records. All operations were performed by a single surgeon (MU) using the same technique except for the first two patients in our series whose arteriotomy was repaired directly. We included symptomatic patients with >70% stenosis and asymptomatic patients with >80% stenosis and excluded patients with severe medical co-morbidities. All the patients underwent a screening Duplex scan of extra-cranial neck vessels to assess the carotid bifurcation stenosis, followed by confirmatory imaging - a combined carotid and coronary angiogram, in our early experience, and noninvasive Magnetic resonance imaging of late. Follow-up clinical assessment was done independently by the vascular surgery and neurology teams, three months and nine months after surgery and annually thereafter. Duplex scan in all and MRA in six patients were employed for check imaging. We assessed these patients for procedure related mortality, ipsilateral cerebral events, and long term patency of Internal carotid artery.

The age range of the patients was 30 to 78 years, with a mean age of 56 years in this cohort that included four women. [Table 1] Thirty seven symptomatic patients with >70% stenosis and two asymptomatic patients with >80% stenosis were included. Two patients with severe coronary artery disease were excluded and referred to an interventional radiologist for carotid artery stenting. One procedure for symptomatic stenosis was performed with synchronous Coronary bypass (CABG).

**Table 1:** Patient characteristics, presentation, co-morbidity and stenosis

Age	Sex	Presentation TIA/RIND/Stroke/AF	Vascular Co-Morbidity	Carotid % Stenosis	Contralateral Carotid Stenosis
58	M	STROKE-MINOR SEQUELAE	NIL	70	NFL
55	M	STROKE-MINOR SEQUELAE	NIL	80	NIL
36	M	STROKE-MINOR SEQUELAE	NIL	80	NIL
64	M	TIA	PVOD	70	50
55	M	TIA	NIL	70	NIL
48	M	ASYMPTOMATIC	PVOD	90	60
63	M	STROKE-MINOR SEQUELAE	PVOD	90	NFL
72	M	TIA	PVOD	70	50
56	M	STROKE-MINOR SEQUELAE	CAD, PVOD	70	100
52	F	STROKE-MINOR SEQUELAE	PVOD, CAD	80	50
39	M	STROKE-MINOR SEQUELAE	PVOD	70	NFL
59	F	STROKE-MINOR SEQUELAE	CAD	9O	70
78	M	STROKE-MINOR SEQUELAE	NIL	70	NIL
46	M	STROKE-MINOR SEQUELAE	CAD-SVD	95	NIL
59	M	STROKE-MINOR SEQUELAE	CAD-TVD, PVOD	90	90
62	M	STROKE-MINOR SEQUELAE	CAD	80	40
47	F	STROKE-MINOR SEQUELAE	NIL	70	NIL
65	M	TIA	CAD-TVD	80	NIL
56	M	RIND	NIL	90	NIL
58	M	TIA	NIL	80	40
58	M	STROKE-MINOR SEQUELAE	CAD	90	NIL
73	M	STROKE-MINOR SEQUELAE	CAD	70	NIL
58	M	STROKE-MINOR SEQUELAE	NIL	90	NIL
39	M	STROKE-MINOR SEQUELAE	PVOD	70	NFL
50	M	STROKE-MINOR SEQUELAE	NIL	80	NIL
69	M	ASYMPTOMATIC	PVOD, CAD	90	30
30	M	STROKE-MINOR SEQUELAE	NIL	70	NIL
58	M	TIA	NIL	80	NIL
70	M	STROKE-MINOR SEQUELAE	NIL	70	50
48	M	STROKE-MINOR SEQUELAE	NIL	80	100
49	M	STROKE-MINOR SEQUELAE	PVOD	80	NIL
57	M	STROKE-MINOR SEQUELAE	NIL	80	NIL
54	M	STROKE-MINOR SEQUELAE	CAD	70	NIL
55	M	STROKE-MINOR SEQUELAE	NIL	90	100
58	M	RIND	NIL	99	30
58	M	TIA	NIL	90	NIL
61	M	STROKE-MINOR SEQUELAE	NIL	80	NIL
58	F	TIA/AF	NIL	80	50
58	M	TIA	NIL	90	NIL

M-Male, F-female, TIA-Transient Ischemic attack, RIND-reversible ischemic neurological deficit CAD-Coronary artery disease(SV-single vessel, DV-double vessel, TD-triple vessel disease), PVOD-Peripheral Vascular occlusive disease AF-amaurosis fugax RAS-Renal artery stenosis, NFL-Non flow limiting WH-wound hematoma

Apart from hypertension (34/39) and smoking (28/39), co-morbidities included diabetes (12/39) and previous myocardial infarction (3/39) [Table 2].

**Table 2:** Co-morbid conditions

Co-morbidity	No. of patients	%
Hypertension	34/39	87
Smoking	28/39	71
Diabetes mellitus	12/39	30
History of myocardial infarction	3/39	7

All the patients underwent the procedure under general anasethesia, with endotracheal intubation, essentially as the surgeon's choice. Triple lumen central venous access, peripheral venous access, radial arterial line and urinary bladder catheterization were standard. The patient was positioned with neck extension, with a roll under scapula, head stabilized on a ring, and with the chin turned to opposite side. Incision was along the anterior border of sternocleidomastoid muscles (SCLM), from the angle of mandible to 5 cm above clavicular head. The carotid sheath was opened and the common carotid artery was dissected and encircled with No:3 silk loop for control. The dissection plane was gently and carefully continued, safeguarding the internal jugular vein anterolaterally in line with SCLM muscle. Gentle dissection was continued upwards, ligating and dividing the common facial vein, and the carotid bifurcation was reached. Taking utmost care to ensure gentle handling, the distal internal carotid artery beyond the disease was dissected and controlled. External carotid and superior thyroid artery were controlled and silk loops placed, leaving the diseased and adherent carotid bifurcation for dissection at a later stage, as the vessels were clamped.

Upon completion of the dissection and control of vessels, Pentothal sodium (5 mg/kg) and methyl prednisolone (30 mg/kg) were administered. Heparin (1 mg/kg) was given intravenously and Common Carotid Artery (CCA), Internal Carotid Artery (ICA), Superficial Temporal Artery (STA) and External Carotid Artery (ECA) were clamped (bulldog clamp for ICA). (Generally, arteriotomy begins 2 cm on the distal CCA, on to the carotid bulb, gradually extending to visualize the atheromatous endpoint in ICA.) The tip of the plaque was released by applying gentle traction, as the plaque was being carefully separated along the endarterectomy plane, till normal endothelium was seen. The proximal end of the plaque was sharply divided using scissors, as low as possible in distal CCA. Having done this, the common carotid artery end of the Pruitt-Inahara shunt was introduced (marked blue) into the CCA, the balloon inflated using 1cc distilled water, obtaining spurting blood at the ICA end. The patient was put in 20 degree Trendelenburg position, the bull dog clamp on ICA released, simultaneously controlling the back bleed from it, and the ICA end of carotid shunt was introduced 2 cm beyond the arteriotomy, inflating its balloon with 0.5 cc of distilled water. The middle, the most adherent part of the plaque, was now dissected out carefully, along with the plaque extension, into the ECA. The endarterectomized artery was then carefully irrigated with heparinised saline and any loose intimal bits gently peeled off or sharply divided. At this time, stump pressure was noted by placing a rubber shod on the CCA end, using an arterial line extension attached to the T segment of the Pruitt Inahara shunt. After recording the stump pressure, continuous perfusion pressure to brain was recorded with the removal of rubber shod. Systemic arterial pressure was monitored via the radial arterial line simultaneously.

An appropriate length of already dissected saphenous vein was excised and the arteriotomy was repaired using two 6/0 polypropylene sutures, commencing at both the ends of the arteriotomy. (Generally, tacking stitches are not required, since the distal end point goes well beyond the plaque and all arterial sutures are taken inside out.) Before completion of suture line, the carotid shunt was removed and clamps reapplied, allowing a good flush of blood from all three carotid arteries. After completion, two to three loops were kept loose to allow deairing. Sutures were hand tied, ECA first and then CCA clamps released, and initial perfusion restarted to ECA. ICA circulation was restored one minute later. Hemostasis was secured and partial heparin reversal with 0.5 mg/kg Protamine was achieved. Lomodex in normal saline was already started at a rate of 20 cc/h, while the clamps were applied. Closed drainage system was instituted and the wound repaired in layers. The patient was then shifted to the ICU, kept on assisted ventilation for four to six hours, to stabilize and to make sure that no neurological deficits existed.

### Surgical Protocol

**Figure 1:**
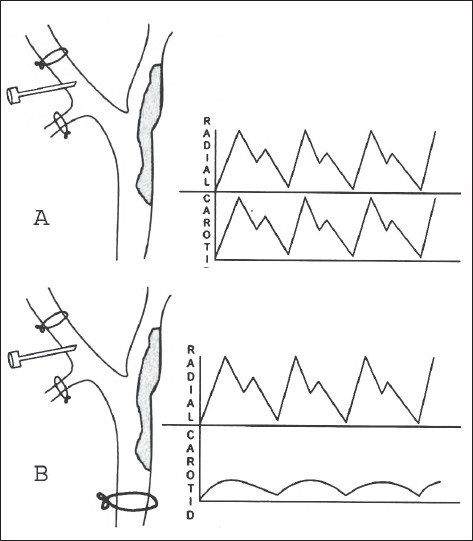
A) Radial and common carotid artery pressures showing equal with identical tracing monitored using needle in ECA after clamping ECA and sup. Thyroid artery B) Stump pressure recording+ monitoring (Greenhalgh's technique) ICA stump pressure appearing dampened upon clamping CCA
- IntraLuminal Shunt-9F Pruitt-Inahara shunt.
- Careful Excision of atheromatous plaque
- Copious irrigation of endarterectomy site with heparinised saline
- Vein Patch Repair
- Systematic sequential declamping & Reperfusion
- Protamine to reverse 50% of initial heparin dose
- Post-op B.P. control to 120 mm Hg systolic (William Baker's recommendation)
- Low mol wt Dextran 20cc/hr for 48 hr
- Ventilation for 4-6hrs to keep PCO2 – 35 mm Hg

- General Anesthesia
- Dissection and control of CCA, ECA, ICA and Sup. Thyroid art
- Heparinisation- 1mg/kg
- Stump Pressure Monitoring (Greenhalgh Technique:[4] [Figure 1]

### Perioperative Pharmacological Strategy

Prior to carotid clamping, 1mg/kg heparin was administered systemically. While the procedure is under way, the anaesthesiologist commenced infusion of Lomodex in saline at 20 cc/h, to prevent platelet aggregation following the procedure. Copious irrigation of endarterrectomy site was done with heparinised saline and any intimal tag was carefully removed.

Following meticulous deairing and reperfusion at the end of procedure, heparin was reversed with Protamines (50% of heparin dose). Eight hours after the surgery, low molecular weight heparin 0.4 ml subcutaneous was administered and continued for seven days. Lomodex infusion was continued for 48 hours. Ecospirin (continued for life long) and clopidogrel (for six months) were added for dual antiplatelet effect. Oral anticoagulation was administered for only one patient who was found to have ulcerated plaques with loose fresh thrombus. Statins and preoperative medications were continued appropriately.

## Results

Of the 39 patients who underwent CEA, there were neither procedure related mortality nor ipsilateral major adverse cerebral events.

### In-Hospital results

One patient, a 58-year-old with diabetes, hypertension and moderate coronary artery disease developed cardiac failure, requiring assisted ventilation, inotropic supports, and prolonged ICU stay. Eventually, the patient recovered fully. Another patient developed postoperative ipsilateral Transient Ischemic Attack (TIA) on the third day, recovering with low molecular weight heparin and dual antiplatelet drugs. Minor morbidities included neck hematoma requiring evacuation in two and transient hypoglossal nerve paresis in three [Table 3].

**Table 3:** Plaque morphology, complications, hospital stay, follow up, comments

Plaque morphology	Peri-Op events	Specific complications	Hosp stay (days)	Follow-up (In months)	Comments
	WH	NIL	7	12 (LFF)	Direct repair
	TIA	NIL	7	48	Direct repair/died(4y)/c/l stoke
	NIL	NIL	7	120	
	NIL	NIL	7	<1	Contralateral massive stroke/death
Ulcerated	NIL	NIL	7	84	
	NIL	NIL	20	96	
	NIL	NIL	7	72	
	NIL	NIL	7	60	Late death due to myocardial infarction
	NIL	NIL	6	60	
Ulcerated	NIL	NIL	7	60	
	NIL	NIL	7	36	
	NIL	CN-XII PARESIS	9	72	
Ulcerated	TIA	Nil	7	48	Underwent stenting/ LFF at 48M
	NIL	NIL	7	36	
	NIL	NIL	7	72	Synchronous CABG
	NIL	NIL	7	60	
	NIL	NIL	6	48	
	NIL	CN-XII PARESIS	6	12	Radiotherapy for tongue cancer
	NIL	NIL	6	48	
	NIL	NIL	5	36	Late death due to myocardial infarction
Ulcerated	NIL	CARDIAC FAILURE	18	36	Prolonged ventilation (cardiac failure)
	NIL	NIL	10	48	
	NIL	NIL	5	12	
	NIL	NIL	7	36	
	NIL	NIL	7	24	
	NIL	NIL	7	24	
	NIL	CN-XII PARESIS	10	24	
	NIL	NIL	6	12	
	NIL	NIL	7	24	
	NIL	NIL	6	16	
	NIL	NIL	6	12	
Ulcerated	NIL	NIL	7	7	
	WH	NIL	9	7	
Ulcerated	NIL	NIL	7	8	
	NIL	NIL	6	6	
Ulcerated	NIL	NIL	6	6	
	NIL	NIL	6	8	
	NIL	NIL	6	4	
	NIL	NIL	6	3	

LFF-Lost for follow-up, CN-Cranial nerve Duplex-N- Normal CABG-Coronary Artery Bypass Grafting, WH- Wound hematoma

### 30-day results

One patient with ulcerated carotid plaque and loose thrombus in ICA developed TIA on the 13^th^ postoperative day, despite oral anticoagulation. The distal end point of vein patch onto ICA was seen to be kinked on DSA, which was successfully managed with carotid stenting. Another patient with bilateral significant carotid disease died of contralateral stroke, while awaiting surgery on the opposite side.

### Late results

One patient who refused treatment for his contralateral disease, succumbed to stroke, four years after primary surgery. Two patients were lost for follow-up. One patient each underwent coronary artery stenting, renal artery stenting and aortofemoral bypass grafting at six months, five years and three years after CEA. Two patients died of coronary artery disease at three years and five years after endarterectomy. The rest of the 33 patients on regular follow-up are keeping good health and enjoying good quality life, totally free of the symptoms pertaining to carotid artery territory [Table 4].

**Table 4:** Serial follow-up depicting stroke free interval and ICA patency

Results	In hospital	30 d	3 m	9 m	1 yr	3yr	4y	5y	6y	7y	8y	10y
Survivors/sample at risk	39/39	38/39	38/38	36/36	30/30	19/20	14/15	9/10	6/6	3/3	2/2	1/1
Major ipsilateral cerebral event/sample at risk	0/39	0/39	0/38	0/36	0/30	0/20	0/15	0/10	0/6	0/3	0/2	0/1
Minor ipsilateral cerebral event/sample at risk	1/39	1/39	0/38	0/36	0/30	0/20	0/15	0/10	0/6	0/3	0/2	0/1
ICA patency by Duplex scan/MRA/sample population	39/39	38/39	38/38	36/36	30/30	20/20	15/15	10/10	6/6	3/3	2/2	1/1

**Figure 2:**
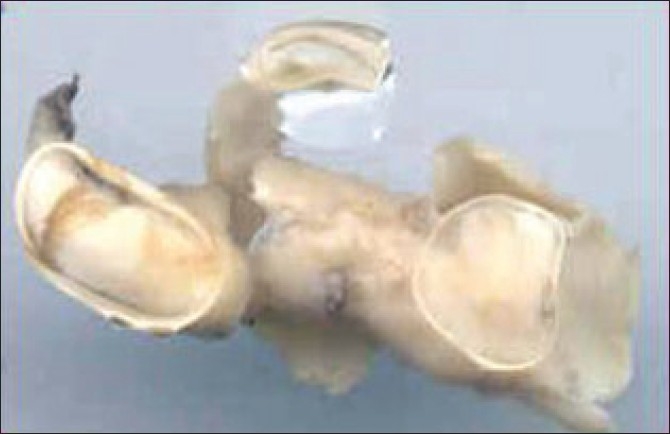
Excised atheromatous carotid plaque in toto

Histopathological examination of excised endarterectomy specimes from carotid artery showed plaques predominantly fibro-mucoid and myxomatous type in most patients and atheromatous type in others [Figure 2].

## Discussion

The primary (principal) endpoint of this retrospective analysis was immediate prevention of stroke in this cohort of 39 patients, who were operated by a single surgeon. The second end point was long-term efficacy towards stroke prevention and to assess the status of the carotid bifurcation - particularly internal carotid artery.

In this study, nine (23%) patients had TIA, one (2.5%) Amaurosis fugax, and two (5%) Reversible Ischemic Neurologic Defect (RIND). A vast majority had developed full-fledged stroke, recovering over time, presenting with minor residual sequalae. The benchmark symptom of carotid bifurcation stenosis was the all too familiar TIA.[8] However just over 20% of the patients are known to present with TIA. A subset may have amaurosis fugax. A good number of patients present with RIND or stroke recovering almost completely or with minor sequelae. Chances of stroke after TIA is 20% in the first week and 35-40% in next three months.[8]

Only two (5%) patients in this cohort were asymptomatic, >80% Internal carotid artery stenosis picked up stenosis on Duplex examination. Asymptomatic carotid artery stenosis, incidentally detected while investigating for atherosclerotic markers like peripheral vascular occlusive disease or coronary artery disease, is generally found only in a small subset of patients in our practice. This trend is at great variance from published Western data, where the asymptomatic group constitutes a large subset.[5–7]

**Figure 3:**
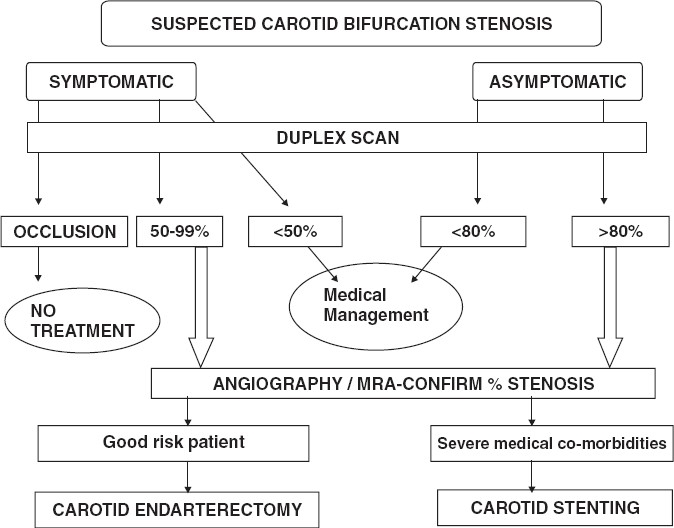
Algorithm for intervention in suspected carotid bifurcation stenosis followed at our institute.(symptomatic >70%, ulcerated plaque >60%, asymptomatic >80% stenosis constituted criteria for CEA in our series)

An algorithm for management options for carotid artery disease is given in [Figure 3]. Duplex scan is universally available and it forms the basic investigating tool.[1,2] We have employed carotid angiogram (DSA) earlier in our experience, but presently obtain an MRA for carotid delineation. Echocardiogram is suggested, to rule out embolic sources from the heart and ascending aorta and also to assess cardiac function. Similarly, contrast coronary angiogram performed for cardiac risk stratification is now done away with and non-invasive assessment is employed, except when serious cardiac symptoms or ECG changes mandate. Patients with more than 70% symptomatic carotid stenosis are selected for endarterectomy.[7,8] However when the duplex scan identifies ulcerated atheromatous plaque, the decision for surgery is made at 60% stenosis, which is based on the fact that atheroembolism leads to serious cerebral outcome than the level of ischemia *per se*. Likewise, asymptomatic stenosis more than 80% warrants intervention in order to escape devastating stroke and its sequelae.[7]

Simultaneous bilateral carotid revascularization is considered hazardous, universally, in view of ensuing intractable cerebral edema. Hence staged procedures are done with symptomatic or high grade stenoses being revascularised first followed by the contralateral side 4-6 weeks later, which is the accepted strategy. However we have not performed bilateral carotid endarterectomy in any of our patients- one patient developed documented contra-lateral stroke two weeks after CEA while waiting for the staged procedure and second patient refused surgery for the contra-lateral disease

Our preference for procedure is under general anaesthesia, which provides unhurried time to perform meticulous and tidy surgery. Well conducted trials - North American Symptomatic Carotid Endarterectomy Trial (NASCET), BIRMINGHAM, and Veteran Administration Symptomatic Carotid Artery Trial - have conclusively proven the efficiency of CEA for stroke prevention but Eversion endarterectomy versus standard carotid endarterectomy (EVEREST) trial mandated patching of the arteriotomy for optimal, immediate and late outcome after endarterectomy.[1,2]

Our policy is to use carotid shunt to provide cerebral circulation while ipsilateral carotid artery is clamped during surgery. Although selective use of shunt is generally the accepted policy in patients with previous stroke, contralateral disease, ICA stump pressure less than 50 mm Hg,[1,2] it is judicious to perfuse the brain during surgery. The intra-operative monitoring using Bispectral Index (BIS) is routinely employed in our centre {which is not specific monitoring}. When the procedure is being performed under general anaesthesia, carotid shunting is necessary, if not mandatory.[6] Hence, we have followed the use of ‘shunt in all’ policy, erring on the safer side, in our institute. In addition, stump pressure is noted and continuous perfusion pressure along with radial artery systemic blood pressure is routinely monitored.

The placement of shunt requires one to three minutes of carotid clamping, which is well-tolerated on keeping satisfactory blood pressure, on 100% oxygen prior to clamping, along with administration of thiopentone sodium to decrease metabolic demand, and methyl prednisolone to stabilise cell membrane. Upon placing the stent, unhurried time is available to complete the removal of plaque, excise endothelial tags and irrigate the denuded endarterectomised media with copious amounts of heparinised saline. With *in situ* shunt, the ICA remains widely open, so that the distal end point of the vein patch is precisely sutured, with no compromise of lumen. On an average, in our practice, 20 minutes of unhurried time and meticulous surgical manoeuvres are required and easily provided when 9F Pruitt-Inahara carotid shunt is used.

Hayes *et al.*[5] from United Kingdom has reported the efficacy of 9F Pruitt Inahara carotid shunt in 548 procedures and found that the mean MCA velocities remain within 10% of preoperative values and that the shunt maintained adequate MCA flow in 98% of the patients during surgery. Above all, reperfusion cerebral edema following endarterectomy is not seen in our patients because of the use of shunt, coupled with control of blood pressure and use of mannitol and steroids for the first two to three post-operative days.

Upon clamping the carotid arteries, our anaesthesiologist commences infusion of low molecular dextran in saline at the rate of 20 cc/h. This helps in preventing platelet aggregation when reperfusion starts following surgery, bringing blood in contact with the endarterectomised ICA, as advised by Richard Royle of Melbourne, by showing abolition of high intensity transient signals (HITS), with the help of trans cranial Doppler.

**Figure 4:**
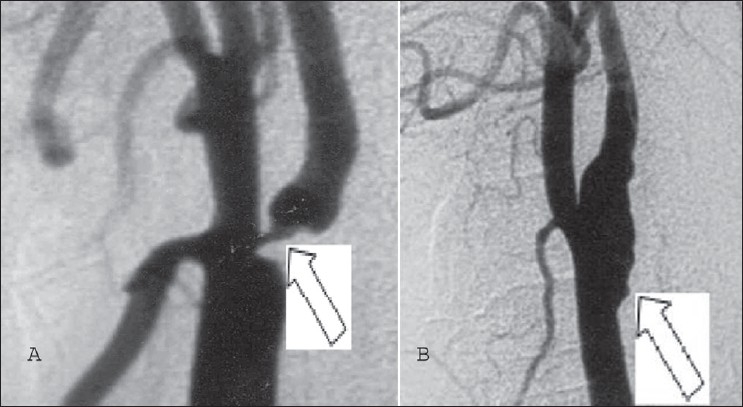
A) Digital subtraction angiogram showing 90% ICA stenosis (white arrow) B) Post. Op DSA showing Vein patch Repair of ICA After excision of carotid Atheromatous Plaque (white arrow)

Current reports have shown conclusive evidence that carotid arteriotomy needs to be repaired using a patch, so as to obviate late stenosis and provide long term excellent outcome following endarterectomy, although earlier literature mentions need for patching only in restenosis and incision taken more than 1cm onto the internal carotid artery. In our experience satisfactory plaque excision required arteriotomy more than 1cm onto ICA and more often than not, ICA was only 4-5mm in size. Prosthetic patch is more expensive and has a higher preponderance for infection; hence only saphenous vein patch was the choice for repair of arteriotomy in our experience.[3] More over autologous vein with viable endothelium not only widens the carotid artery but also provides thrombo-resistant tissue in the endarterecomised carotid avenue.[11,12] This particular attention to vein patch repair has helped prevent early post-operative ICA thrombosis as well has achieved long term ICA patency consistently [Figure 4].

**Figure 5:**
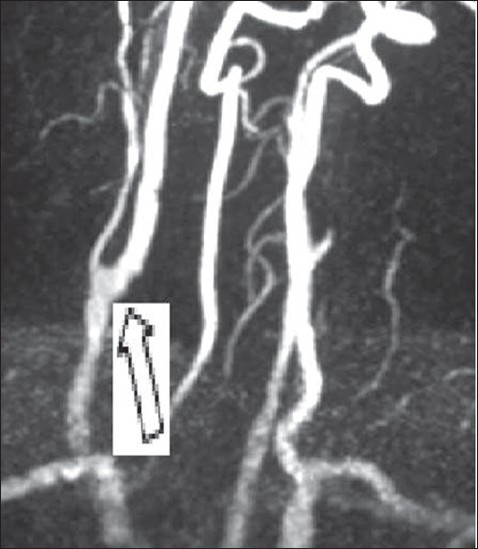
MRA showing normalized ICA at 9yrs after CEA For 70% stenosis with contra lateral ICA occlusion

Incidence of peri-operative stroke is reported to be 0.5-5% in literature.[1,2,6,11,12] Mortality following CEA is predominantly due to cardiac events and rarely due to adverse cerebral events. In our series all patients survived and none developed major cerebral adverse events. Two patients developed TIA- one patient on 2^nd^ post-operative day, managed medically and the other on the 13^th^ post-operative day requiring carotid artery stenting for correction of kink at the distal endpoint of ICA patch. Long term re-stenosis was reported to be 2-15% till regular patching of the arteriotomy reduced it to 3-5% when prosthetic or bovine pericardial patch was used.[11] Although, patient population is small, vein patch arteriotomy repair in our series has shown excellent long term patency of ICA and near total abolition of major ipsi-lateral cerebral events [Figure 5].

Incidence of stroke is estimated to be 60 in 100000 in general population. Carotid plaque as the culprit lesion for adverse cerebral events is far less prevalent in India than in the western world. It is a fact that due to the absence of screening programmes and general unawareness in our society, we see more of established strokes. With the universal availability and application of ultrasound and duplex scan, more and more patients are being evaluated for treatable causes of stroke.

Our experience has also shown that associated intra-cranial lesions and internal carotid artery dissection are more prevalent in our patients. Carotid atheroma has more potential to lead to atheroembolism, with resultant adverse cerebral events, than cerebral hypoperfusion, due to significant stenosis. Currently carotid endarterectomy and stenting with protective device is increasingly being undertaken, with good success rate in many centres across our country.

Carotid endarterectomy has a proven track record for its efficacy to prevent stroke and is the most critically evaluated and scrutinized procedure in the literature with North American Symptomatic Carotid Endarterectomy Trial (NASCET), European Carotid Surgery Trial (ECST), and Veteran administration trials. Asymptomatic Carotid Trial provided conclusive evidence that patients with more than 80% asymptomatic stenosis immensely benefitted from surgery. Although carotid stenting is evolving into an effective procedure in preventing stroke, current evidences have supported superiority of endarterectomy in prospective as well as retrospective studies.[9] However, stenting is the management option in restenosis and in conditions of ‘hostile neck’ following radiation or radical neck dissection. The prospect of re-occlusion following stenting and the increased cost involved in stenting with protection devices, makes carotid endarterectomy all the more the primary therapeutic modality for patients with high grade carotid stenoses, in our practice. Currently, ongoing Prospective Trans Atlantic Asymptomatic Carotid intervention Trial II (TACIT II) in 150 centres involving 3500 patients with three arms -- optimal medical management, carotid stenting with protective devices and carotid endarterectomy -- is likely to provide the best therapeutic option for patients with significant carotid artery disease.[10]

**Figure 6:**
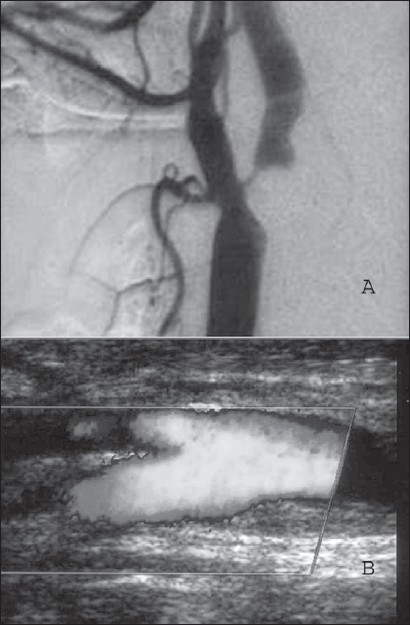
A) DSA showing > 95 % ICA stenosis B) colour Duplex scan showing widely opened up ICA ---- 2 yrs post. op of 58 yr old symptomatic patient shown in Fig 4a

This retrospective study has shown that freedom from stroke both at short term and long term intervals is comparable to results reported in world literature.[1,2,6,9] Meticulous surgical technique with gentle manipulation of the carotid vessels and use of carotid shunt provided unhurried time for the procedure. Maintaining mean MCA blood flow in Pruitt-Inahara shunt[5] has also helped in repairing the arteriotomy using a vein patch. Endarterectomised artery is thrombogenic and hence its luminal widening using viable autologous saphenous vein patch not only helps avoid high intensity transit signals (HITS) resulting in unobstructed blood flow across but also keeps the reconstructed ICA patent providing durable results in our study [Figure 6].

## Summary

We report a retrospective study of 39 patients who underwent consecutive carotid endarterectomy at our institute. Our protocol for imaging was Duplex scan and MRA for the culprit lesion and non-invasive tests for cardiac risk assessment. Our standardized surgical strategy consisted of general anaesthesia, carotid shunting and vein patch arteriotomy repair. Short term results and long term follow-up showed excellent results towards prevention of stroke with quality of life.

## Conclusion

The bench mark procedure of CEA is being underutilized in our country, considering the vast number of patients who report with established stroke as a sequalae to carotid bifurcation stenosis. There is a great need to screen those at increased vascular risk, over 55 years, particularly patients presenting with atherosclerotic expression in vascular territories elsewhere.

The purpose of this report - a torchbearer article dedicated to the invaluable procedure of carotid endarterectomy - is to underline and propagate safety and utility of this procedure in appropriate patients, to physicians and neurologists. Timely referral of those patients with symptomatic >70% and asymptomatic >80% carotid stenosis would prevent stroke and restore quality of life with the judicious use of Carotid Endarterctomy.
